# Lidocaine for postoperative pain after cardiac surgery: a systematic review

**DOI:** 10.1186/s13019-021-01549-0

**Published:** 2021-05-31

**Authors:** Michael R. Boswell, Rajat N. Moman, Melissa Burtoft, Harrison Gerdes, Jacob Martinez, Danielle J. Gerberi, Erica Wittwer, M. Hassan Murad, W. Michael Hooten

**Affiliations:** 1 Department of Anesthesiology and Perioperative Medicine, Mayo Clinic, 200 First St SW, Rochester, MN 55902 USA; 2 Mayo Clinic Library, Mayo Clinic, Rochester, MN USA; 3 Division of Preventative Medicine, Department of Internal Medicine, Mayo Clinic, Rochester, MN USA; 4 Division of Pain Medicine, Mayo Clinic, Rochester, MN USA

**Keywords:** Systematic review, Lidocaine, Meta-analysis, Cardiac surgery, Postoperative pain

## Abstract

**Objective:**

Lidocaine is one of the most widely used local anesthetics with well-known pharmacological properties. The purpose of this systematic review is to investigate the effects of lidocaine on postoperative pain scores and recovery after cardiac surgery.

**Methods:**

A comprehensive database search was conducted by a reference librarian for randomized clinical trials (RCT) from January 1, 1980 to September 1, 2019. Eligible study designs included randomized controlled trials of lidocaine for postoperative pain management in adults undergoing cardiac surgery. After removal of duplicates, 947 records were screened for eligibility and 3 RCTs met inclusion criteria.

**Results:**

Sources of bias were identified in 2 of 3 RCTs. Lidocaine was administered intravenously, topically, and intrapleurally. Key findings included [1] 2% lidocaine placed topically on chest tube prior to intraoperative insertion was associated with significantly lower pain scores and lower cumulative doses of fentanyl; and [2] 2% lidocaine administered intrapleurally was associated with significantly lower pain scores and significant improvements in pulmonary mechanics. Lidocaine infusions were not associated with significant changes in pain scores or measures of recovery. No significant associations were observed between lidocaine and overall mortality, hospital length of stay or ICU length of stay. No data were reported for postoperative nausea and vomiting or arrhythmias.

**Conclusions:**

Due to the favorable risk profile of topical lidocaine and the need for further advancements in the postoperative care of adults after cardiac surgery, topically administered lidocaine could be considered for incorporation into established postoperative recovery protocols.

**Supplementary Information:**

The online version contains supplementary material available at 10.1186/s13019-021-01549-0.

## Introduction

Cardiac surgery, compared to other major surgical procedures, is associated with considerable pain [1], and research aimed at optimizing postoperative pain management is ongoing [2]. Pain after cardiac surgery is most severe on postoperative day (POD) 1 and 2 [3, 4]. Although pain diminishes throughout the postoperative period, nearly half of patients continue to report severe pain at rest on POD 4 [5]. The location of pain changes throughout the postoperative period. During the early postoperative course, the majority of severe pain involves the primary operative site but, after POD 2, other important areas of pain include the lower extremities, due to vein extraction, and shoulders [4, 5]. Coughing, moving, turning, deep breathing and using incentive spirometry are associated with pain through POD 4 and POD 6 [3, 5]. Effective management of acute postoperative pain is important because greater acute pain severity during the postoperative period is associated with development of chronic postoperative pain [6, 7].

In the Enhanced Recovery After Surgery Society recommendations for perioperative cardiac surgery care, multimodal opioid-sparing pain management plans are strongly recommended (Class I recommendation) [8]. Multimodal pain management after cardiac surgery frequently incorporates local anesthetics administered intravenously, infiltrated perineurally, or infused into the epidural space [9]. Lidocaine is widely used in the perioperative period but, unlike other local anesthetic drugs, topical formulations of lidocaine are readily available and it can be safely administered as an intravenous infusion which expands the therapeutic uses of this particular drug. However, the effects of lidocaine on postoperative pain and recovery after cardiac surgery have not been systematically reviewed. Thus, the primary objective of this systematic review is to investigate the effects of lidocaine on postoperative pain scores after cardiac surgery. Secondary objectives include investigating the effects of lidocaine on postoperative opioid consumption, rate of postoperative nausea and vomiting, and hospital length of stay. In addition, adverse effects associated with lidocaine, including arrhythmias and all-cause mortality, will be documented.

## Methods

### Study protocol

Preferred Reporting Items for Systematic Reviews and Meta-Analyses guidelines [10] were followed. An a priori protocol was followed. The trial was registered in the PROSPERO database (CRD42020152017) [11].

### Search strategy

The medical literature was searched by a medical reference librarian for content about the postoperative effects of lidocaine after cardiac surgery. The search strategies were created using a combination of keywords and standardized indexing terms. A comprehensive search of databases from January 1, 1980 to September 1, 2019 was conducted. Manuscripts were restricted to the English language. Searches were executed in ClinicalTrials.gov, Ovid EBM Reviews, Ovid Embase, Ovid Medline, Scopus, and Web of Science. Search strategies are presented in Additional file 1: Appendix A.

### Study selection process

Study inclusion criteria included (1) randomized clinical trials (RCTs); (2) studies that assessed postoperative pain and recovery in patients after cardiac surgery; (3) studies that assessed the use of topical, intravenous, or other routes of lidocaine administration; (4) studies from 1980 to present day; and (5) studies in the English language. Exclusion criteria included (1) studies of non-cardiac surgery patients; and (2) non-human studies.

Two independent pairs of reviewers screened all titles and abstracts identified by the search strategy in the first review phase. In the second review phase, the two pairs of independent reviewers screened the full text of all studies identified in the first phase and applied inclusion and exclusion criteria. No disagreements on inclusion were observed during study selection.

### Data extraction

Data were extracted by four independent reviewers using a templated electronic database. Data abstracted included postoperative pain scores, postoperative opioid consumption, postoperative nausea and vomiting, and hospital length of stay. Lidocaine side effects were also abstracted including arrhythmias and all-cause mortality.

### Risk of Bias assessment

Study quality was assessed using Cochrane’s Risk of Bias tool (RoB2) and the assessment was reported as an overall risk of bias [12]. The overall risk of bias using RoB2 is judged to be “low risk of bias,” “some concerns for bias,” or “high risk of bias.” [12] Reviewer discrepancy was resolved by consensus or by a third reviewer. Certainty in the estimates of the effect of lidocaine on pain was evaluated using the GRADE approach [13] adapted for use with quantitative data that are not combinable in meta-analysis [14].

### Evidence synthesis

Due to heterogeneity in study characteristics, settings, and outcomes, a meta-analysis was not feasible; thus, the results are presented using a narrative approach. A narrative approach can be used when a content area has been studied using disparate methods and the outcomes were variable [15, 16]. This approach is useful when key clinical factors vary between studies. Narrative methods for evidence synthesis have been used to study various populations of patients with pain [17–22].

## Results

### Characteristics of included studies

**Fig. 1:**
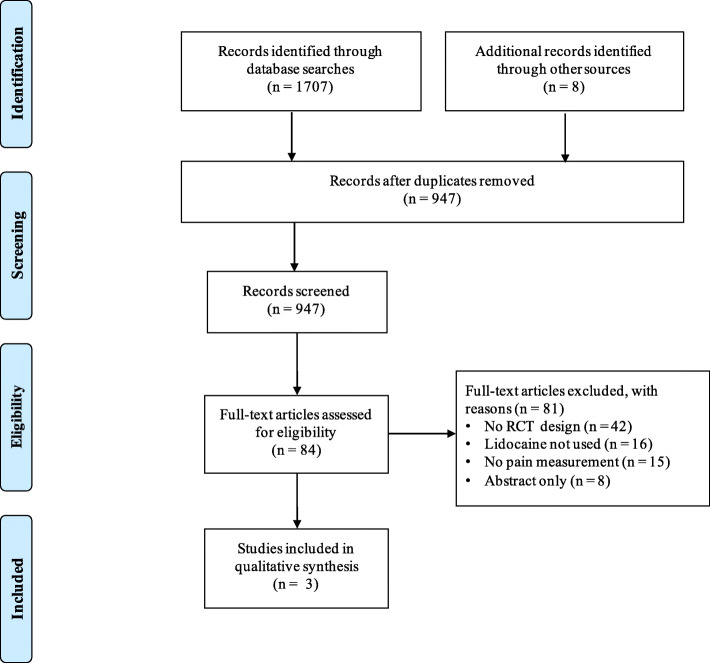
Preferred reporting items for systematic reviews and meta-analyses flow chart of the study selection process. Note: Reproduced from Moher D, Liberati A, Tetzlaff J, Altman DG, Group P. Preferred reporting items for systematic reviews and metaanalyses: the PRISMA statement. BMJ. 2009;339:b2535 [10]

A flow diagram of the study selection process is depicted in Fig. 1. Three studies [23–25] met inclusion and exclusion criteria (Table 1), and were included in the qualitative analysis. All studies were RCTs involving patients who underwent a median sternotomy for coronary artery bypass graft surgery. The route of lidocaine administration in these studies included an intravenous infusion [23], topical application [24] and intrapleural injection [25]. The primary outcome for each of these studies was postoperative pain scores measured using a visual analogue scale (VAS) or numeric rating scale (NRS) assessed at time intervals ranging from 4 h to 4 days postoperatively. Studies that used a VAS presented patients with a 10 cm visual scale labeled either 0 to 10 [23] or 0 to 100 [24]; a score of 0 denoted no pain and the maximum scores of 10 or 100 denoted the worst possible pain. In the study that used the NRS [25], pain was rated on a scale between 0 to 10 where 0 denoted no pain and 10 denoted the worse possible pain. One study [23] reported postoperative pain scores at hours 4, 8 and 16. All three studies [23–25] reported postoperative pain scores at hours 24 and 48. Two studies [23, 24] reported mortality data and one study [23] reported intensive care unit and hospital length of stay. Data about postoperative nausea and vomiting, and arrhythmias were not reported. Other outcomes varied between studies and included (1) cumulative postoperative opioid dose, (2) benzodiazepine dose; (3) beta-blocker dose; (4) frequency of patient controlled analgesia (PCA) button pushes; (5) proportion of patients reporting chest tubes as the most painful site; (6) postoperative day of chest tube removal; (7) mean forced expiratory volume at 1 sec (FEV1) on POD 1; (8) sedation scores; (9) occurrence of postoperative myocardial infarction; and (10) time to extubation.

**Table 1:** Study characteristics

Author	Study design	Participants	Intervention vs. control	Timeline	Primary outcome	Secondary outcome	Follow-up period	Drop outs	Risk of bias
Insler, 2009 [23]	Randomized double-blind placebo-controlled trial	*n* = 100; MS for CABG; mean age 63 years; 72% male	IV lidocaine infusion (*n* = 44) vs. placebo substitute (*n* = 45)	Anesthesia induction until either ICU dismissal or PO hour 48	VAS pain score at PO hours 4, 8, 16, 24, 48, 96	ICU hemodynamics; MI; extubation time; sedation score; PO fentanyl, midazolam, propranolol doses; ICU and hospital length of stay	ICU dismissal or PO hour 48	11	Some concerns
Kang, 2014 [24]	Randomized placebo-controlled trial	n = 45; MS for CABG, mean age 68 years; 67% male	Topical lidocaine 2% gel on chest tubes (*n* = 22) vs. normal saline placebo (*n* = 23)	Single application with intraoperative insertion of chest tubes	VAS pain scores at extubation and POD 1, 2, 3, 7	Cumulative PCA fentanyl PCA; number of PCA button pushes; chest tube worse site of pain	POD 7	3	Low
Mashaqi, 2018 [25]	Randomized, double-blind placebo-controlled trial	*n* = 40; MS for CABG; age and sex not reported	Intrapleural 12 mL 2% lidocaine (n = 20) vs. 12 mL 0.9% saline solution placebo (*n* = 20)	Single injection on POD 1 and 2; administered via left-sided double-lumen chest tube	NRS pain scores on POD 1 and POD 2	FEV1 before and after injections on POD 1 and POD 2	POD 2	0	High

*CABG* coronary artery bypass graft, *MS* median sternotomy, *IV* intravenous, *ICU* intensive care unit, *MI* myocardial infarction, *POD* postoperative day, *PO* postoperative, *VAS* visual analog scale, *NRS* numerical analog scale, *PCA* patient controlled analgesia, *FEV1* forced expiratory volume at one second

### Risk of Bias evaluation

The RoB2 was used to assess bias in the 3 studies. One study demonstrated a high risk of bias [25], one study had some concerns for bias [23], and one study had a low risk of bias [24]. In the study that had a high risk of bias [25], information about the method of randomization or blinding were not included. In the study that had some concerns for bias [23], potential reporting biases were identified because greater than 10% of patients were excluded for protocol deviations, excessive bleeding, supratherapeutic lidocaine serum levels, and use of multiple ionotropic medications.

### Pain intensity

All three studies reported pain intensity using the VAS [23, 24] or NRS [25]. Postoperative pain scores were reported across a range of time intervals spanning 4 h to 4 days.

In the Insler et al. study [23] patients were randomized to receive an intravenous lidocaine or placebo infusion from induction of general anesthesia to postoperative hour 48 or intensive care unit (ICU) dismissal, whichever came first. The VAS was assessed at hours 4, 8, 16, 24, 48, and 96 after ICU admission. No statistically significant difference in pain scores were observed between the lidocaine and control groups [23]. The certainty in evidence was very low due to severe imprecision.

In the Kang et al. study [24], patients were randomized to receive an application of topical 2% lidocaine gel or normal saline as a placebo on chest tubes prior to intraoperative placement. At the time of extubation and on POD 1, 2 or 3 patients in the lidocaine group reported significantly lower chest tube-related pain scores compared to the placebo group. Similarly, at the time of extubation and on postoperative day 7, the proportion of patients who reported the chest tube site as the “most painful site” was significantly less in the lidocaine group compared to the placebo group. The certainty in evidence was low due to imprecision.

In the Mashaqi et al. study [25], patients were randomized to receive 12 ml of 2% lidocaine intrapleurally or a saline solution placebo on POD 1 and 2. Mean pain scores were significantly different pre- and post-intrapleural injection in the lidocaine group compared to the placebo group on POD 1 and 2. The certainty in evidence was very low due to severe imprecision.

### Opioid-sparing effects

Two studies [23, 24] reported the cumulative opioid dose during the postoperative hospital stay. Insler et al. [23] reported the cumulative postoperative fentanyl dose administered via a nurse-driven protocol. No significant group difference in cumulative fentanyl dose was observed between the lidocaine and placebo groups.

Kang et al. [24] reported the cumulative postoperative fentanyl dose administered via PCA and the number of PCA button pushes was also recorded. The cumulative fentanyl dose was significantly lower in the group that received topical lidocaine on chest tubes prior to insertion compared to placebo. Similarly, the number of PCA button pushes was significantly lower in the group that received topical lidocaine compared to placebo.

### Mortality and hospital length of stay

Two studies reported mortality data. Insler et al. [23] reported one death in the lidocaine infusion group that was determined not to be related to lidocaine, and Kang et al. [24] reported no deaths attributed to “surgical mortality.” In the Insler et al. [23] study, data were reported about ICU and hospital length of stay but no between-group differences in either measure were observed.

### Forced expiratory volume at 1 s

In the Mashaqi et al. [25] study, patients randomized to the intrapleural lidocaine group experienced statistically significant improvements in FEV1 compared to placebo on POD 1 and 2.

## Discussion

The key findings of this systematic review include (1) 2% lidocaine placed topically on chest tubes prior to intraoperative insertion was associated with significantly lower pain scores and lower cumulative doses of fentanyl (certainty in evidence low); and (2) 2% lidocaine administered intrapleurally was associated with significantly lower pain scores and significant improvements in FEV1 (certainty in evidence very low). However, lidocaine infusions were not associated with significant changes in pain scores, and no significant associations were observed between postoperative lidocaine use and overall mortality, hospital length of stay or ICU length of stay (certainty in evidence very low). No data were reported for other secondary outcome measures including postoperative nausea and vomiting or arrhythmias.

The results of this study suggest that the pharmacology of topical lidocaine warrants further consideration. Lidocaine contains an amide and tertiary amine group, and the pKa is 7.9 [26]. At equilibrium, lidocaine exists as a positively charged cation and an uncharged free base. Basic conditions increase the proportion of free base that exists in solution; this is important because the free base of lidocaine penetrates the lipid containing layer of outer skin, the stratum corneum, and mucosal membranes [27]. The mean depth of skin penetration following topical application of a lidocaine and prilocaine mixture is 5 to 6 mm [28, 29], but the depth of mucosal penetration remains undetermined. The local anesthetic effects of lidocaine are produced predominately by blockade of voltage-gated sodium channels which inhibit neuronal depolarization, and the formation and propagation of action potentials [26]. In addition to the direct analgesic effects on sodium channels, lidocaine has anti-inflammatory effects. In preclinical [30–33] and clinical studies [34, 35], lidocaine reduces macrophage activation and recruitment; blocks production of tumor necrosis factor and interleukin-6; and inhibits prostaglandin, thromboxane, and histamine release. The combined local anesthetic and anti-inflammatory effects of lidocaine provide an underlying pharmacological mechanism for the clinical effects observed in the Kang el al [24]. and Mashaqi et al. [25] studies.

Lidocaine infusions have been associated with improvements in postoperative pain, reduced postoperative opioid use, reduced incidence of nausea and vomiting, and reduced length of hospital stay in patients after non-cardiac surgery [36–38]. However, these favorable postoperative outcomes were not reproduced in the Insler et al. [23] study despite recruitment of an appropriately powered cohort, use of adequate lidocaine dosages for a sufficient time period, and documentation of therapeutic lidocaine serum levels. One possible explanation for these contrasting findings is the systemic inflammatory response produced by cardiopulmonary bypass [39]. This is an important consideration because cardiopulmonary bypass clearly distinguishes cardiac surgery from other major surgical procedures. Following intravenous administration, 70% of lidocaine is metabolized to N-ethylglycine [40] which is a competitive inhibitor of glycine transporter 1 [41]. This transporter regulates extracellular glycine which is the main inhibitory neurotransmitter in the spinal cord and brain stem [42]. Inhibition of glycine transporter 1 by N-ethylglycine leads to increases in serum and cerebrospinal fluid glycine concentrations, and is associated with anti-nociceptive effects in preclinical studies [41, 43]. More specifically, following systemic administration in an experimental pain model, N-ethylglycine attenuates acute inflammatory hyperalgesia [43]. However, the effects of N-ethylglycine on glycine transporter function may be disrupted by the physiological responses to cardiopulmonary bypass. In preclinical studies, intracerebral glycine concentrations were significantly elevated above baseline levels during (1) hypothermic circulatory arrest; (2) cardiopulmonary bypass reperfusion; and (3) glycine levels remained elevated for 2 to 8 h after hypothermic circulatory arrest [44–46]. Thus, in patients undergoing cardiac surgery, a key mechanism responsible for the analgesic effects of intravenous lidocaine infusions may be disrupted by the abrupt changes in glycine concentrations that occur in response to cardiopulmonary bypass.

The findings of this systematic review have implications for clinical practice and future research. First, the postoperative benefits and low risk profile of topical lidocaine suggests that intrapleural lidocaine and placement of lidocaine gel on chest tubes could be incorporated into established recovery protocols [8]. Second, topical lidocaine was associated with improvements in pain and recovery, but the findings need to be replicated in future RCTs. Third, although intravenous lidocaine infusions were not associated with significant changes in postoperative pain and recovery, future RCTs should be considered for patients receiving cardiac surgery that does not require cardiopulmonary bypass.

This review has limitations. Although a comprehensive search strategy identified 947 records, only 3 RCTs met criteria for inclusion. Thus, additional RCTs of lidocaine for postoperative pain and recovery after cardiac surgery are needed. Two of three RCTs were found to be at risk of bias [23, 25], and the certainty in evidence for all RCTs ranged from low to very low. Consequently, the results of this systematic review should be interpreted in light of the identified risk of bias, and the low to very low certainty in evidence of the three RCTs that met our inclusion and exclusion criteria. Finally, no data were reported for important secondary outcome measures including postoperative nausea and vomiting, or arrhythmias.

## Conclusions

In conclusion, although topical lidocaine was associated with improvements in postoperative pain and measures of recovery, the results of these RCTs need to be replicated in in future clinical trials. Lidocaine infusions have proven benefits for patients after non-cardiac surgery but ongoing work is needed to understand the potential impact that cardiopulmonary bypass has on the postoperative effects of lidocaine infusions. These results should be interpreted with full knowledge of the risk of bias, and the low to very low certainty in evidence that characterize the RCTs comprising this systematic review. However, due the favorable risk profile of topical lidocaine and the need for further advancements in the postoperative care of adults after cardiac surgery [47], topically administered lidocaine could be considered for incorporation into established postoperative recovery pathways.

## Supplementary Information

**Additional file 1.**

## Data Availability

No supporting data to report.

## Abbreviations

POD: Postoperative day
RCT: Randomized clinical trial
RoB2: Cochrane’s Risk of Bias tool
VAS: Visual analogue scale
NRS: Numeric rating scale
PCA: Patient controlled analgesia
FEV1: Forced expiratory volume at one second
ICU: Intensive care unit
