# Supplementary material for: Lidocaine for postoperative pain after cardiac surgery: a systematic review
Source: J Cardiothorac Surg. 2021 May 31;16:157. doi: 10.1186/s13019-021-01549-0 (PMC8166031; doi:10.1186/s13019-021-01549-0)
Supplement: Supplementary file 1 — Additional file 1. [file 13019_2021_1549_MOESM1_ESM.docx]

**APPENDIX A**

**ClinicalTrials.gov**

lidocaine | "cardiac surgery" OR "heart surgery" OR sternotomy | Adult

**EBM Reviews:**

(("lidocaine" or "2-(diethylamino)-n-(2,6-dimethylphenyl)acetamide" or "2-2etn-2mephacn" or "2 diethylamino 2',6' acetoxylidide" or "aeroderm" or "akten" or "alpha diethylamino 2,6 dimethylacetanilide" or "alphacaine" or "anestacaine" or "anestacon*" or "aritmal" or "astracaine" or "betacaine" or "cidancaina" or "corus 1030" or "corus1030" or "cuivasil" or "dalcaine" or "dentipatch" or "dequaspray" or "diethylamino 2,6 dimethylacetanilide hydrochloride" or "diethylaminodimethylacetanilid*" or "dolicaine" or "dube spray" or "duncaine" or "dynexan" or "ela-max" or "esracain" or "farmacaina" or "gesicain" or "glydo" or "gravocain" or "isicaine" or "jetokain" or "l-caine" or "lecasin" or "leostesin" or "lida mantle" or "lidocaton" or "lidocor" or "lidocorit" or "lidoderm" or "lidoject" or "lidonest" or "lidopain" or "lidopen" or "lidothesin" or "lignocaine" or "lignostab" or "lincaine" or "liquocaine" or "maricaine" or "n,n diethyl n' (2,6 dimethylphenyl)glycinamide" or "neo novutox" or "neolidocaton" or "omega diethylamino 2,6 dimethylacetanilide" or "octocaine" or "otipax" or "remicaine" or "restylane lyft" or "roxicaina" or "rucaina" or "ruciana" or "solarcaine" or "solcaine" or "truxacaine" or "uad caine" or "vasocaine" or "versatis" or "xidocaine" or "xiline" or "xilocaina" or "xilonest" or "xilotane" or "xilyne" or "xylcaine" or "xylestesin" or "xylesthesin" or "xylocain*" or "xylocard" or "xylocitin" or "xyloctin" or "xyloneural" or "xylonor" or "xyloproct" or "xyloton" or "xylotox" or "xylyne" or "zingo" or "ztlido").ab,kf,ti.) AND ((cardiosurg* or ((aortocoronary or cardi* or intracardi* or pericardi* or coronary or heart or myocardi* or transmyocardi* or valv* or internal-mammary) adj3 (anastomosis or annul* or bypass* or resection or revasculari* or surg* or operation* or recanali* or shunt* or reperfusion* or stenting or aneurysmectom* or massage or graft* or transplant* or allograft* or heterograft* or allotransplant* or heterotransplant* or homograft* or homotransplant* or xenotransplant* or implant* or atherectom* or balloon-dilation)) or sternotom* or sternum-osteotom* or ((atrial or arterial or double) adj1 switch) or jatene or rastelli or senning or atrial-switch or mustard or cardiomyoplast* or annuloplast* or (valve adj2 annul*) or angioplast* or vineberg or fontan or hemi-fontan or norwood or glenn or ((bidirectional or bi-directional or cavopulmonary) adj2 (shunt* or anastomos*)) or pericardiostom* or pericardial-window or pericardiectom* or pericardotom* or pericardiotom* or pericardiocentesis or pericardicentesis or (pericardi* adj2 (aspirat* or punctur*)) or ((valv* or bicuspid or tricuspid) adj2 (bioprosthe* or prosthe* or replac* or artificial or repair or reconstruct*)) or valvuloplast* or valvulotom* or valvotom* or commissurotom* or ross).ab,kf,ti.) AND ((postoperative* or post-operative* or post-op* or postsurg* or post-surg* or post-procedur* or postprocedur* or postanesth* or postanaesth* or post-anesth* or post-anaesth* or PONV).ab,kf,ti. OR ("length of stay" or "hospital stay*" or "stay length*" or LOS).ab,kf,ti.) Limit to 1980+

**Embase (1974+)**

((exp lidocaine/ OR (("lidocaine" or "2-(diethylamino)-n-(2,6-dimethylphenyl)acetamide" or "2-2etn-2mephacn" or "2 diethylamino 2',6' acetoxylidide" or "aeroderm" or "akten" or "alpha diethylamino 2,6 dimethylacetanilide" or "alphacaine" or "anestacaine" or "anestacon*" or "aritmal" or "astracaine" or "betacaine" or "cidancaina" or "corus 1030" or "corus1030" or "cuivasil" or "dalcaine" or "dentipatch" or "dequaspray" or "diethylamino 2,6 dimethylacetanilide hydrochloride" or "diethylaminodimethylacetanilid*" or "dolicaine" or "dube spray" or "duncaine" or "dynexan" or "ela-max" or "esracain" or "farmacaina" or "gesicain" or "glydo" or "gravocain" or "isicaine" or "jetokain" or "l-caine" or "lecasin" or "leostesin" or "lida mantle" or "lidocaton" or "lidocor" or "lidocorit" or "lidoderm" or "lidoject" or "lidonest" or "lidopain" or "lidopen" or "lidothesin" or "lignocaine" or "lignostab" or "lincaine" or "liquocaine" or "maricaine" or "n,n diethyl n' (2,6 dimethylphenyl)glycinamide" or "neo novutox" or "neolidocaton" or "omega diethylamino 2,6 dimethylacetanilide" or "octocaine" or "otipax" or "remicaine" or "restylane lyft" or "roxicaina" or "rucaina" or "ruciana" or "solarcaine" or "solcaine" or "truxacaine" or "uad caine" or "vasocaine" or "versatis" or "xidocaine" or "xiline" or "xilocaina" or "xilonest" or "xilotane" or "xilyne" or "xylcaine" or "xylestesin" or "xylesthesin" or "xylocain*" or "xylocard" or "xylocitin" or "xyloctin" or "xyloneural" or "xylonor" or "xyloproct" or "xyloton" or "xylotox" or "xylyne" or "zingo" or "ztlido").ab,kw,ti.) AND (exp heart surgery/ or exp sternotomy/ OR (cardiosurg* or ((aortocoronary or cardi* or intracardi* or pericardi* or coronary or heart or myocardi* or transmyocardi* or valv* or internal-mammary) adj3 (anastomosis or annul* or bypass* or resection or revasculari* or surg* or operation* or recanali* or shunt* or reperfusion* or stenting or aneurysmectom* or massage or graft* or transplant* or allograft* or heterograft* or allotransplant* or heterotransplant* or homograft* or homotransplant* or xenotransplant* or implant* or atherectom* or balloon-dilation)) or sternotom* or sternum-osteotom* or ((atrial or arterial or double) adj1 switch) or jatene or rastelli or senning or atrial-switch or mustard or cardiomyoplast* or annuloplast* or (valve adj2 annul*) or angioplast* or vineberg or fontan or hemi-fontan or norwood or glenn or ((bidirectional or bi-directional or cavopulmonary) adj2 (shunt* or anastomos*)) or pericardiostom* or pericardial-window or pericardiectom* or pericardotom* or pericardiotom* or pericardiocentesis or pericardicentesis or (pericardi* adj2 (aspirat* or punctur*)) or ((valv* or bicuspid or tricuspid) adj2 (bioprosthe* or prosthe* or replac* or artificial or repair or reconstruct*)) or valvuloplast* or valvulotom* or valvotom* or commissurotom* or ross).ab,kw,ti.) AND (exp postoperative complication/ or exp postoperative period/ OR ((postoperative* or post-operative* or post-op* or postsurg* or post-surg* or post-procedur* or postprocedur* or postanesth* or postanaesth* or post-anesth* or post-anaesth* or PONV).ab,kw,ti.) OR exp "length of stay"/ OR (("length of stay" or "hospital stay*" or "stay length*" or LOS).ab,kw,ti.)) NOT (exp child/ not exp adult/, exp animal/ not exp human/) Limit to 1980+

**Ovid MEDLINE(R) 1946 to Present and Epub Ahead of Print, In-Process & Other Non-Indexed Citations and Ovid MEDLINE(R) Daily:**

((exp Lidocaine/ OR (("lidocaine" or "2-(diethylamino)-n-(2,6-dimethylphenyl)acetamide" or "2-2etn-2mephacn" or "2 diethylamino 2',6' acetoxylidide" or "aeroderm" or "akten" or "alpha diethylamino 2,6 dimethylacetanilide" or "alphacaine" or "anestacaine" or "anestacon*" or "aritmal" or "astracaine" or "betacaine" or "cidancaina" or "corus 1030" or "corus1030" or "cuivasil" or "dalcaine" or "dentipatch" or "dequaspray" or "diethylamino 2,6 dimethylacetanilide hydrochloride" or "diethylaminodimethylacetanilid*" or "dolicaine" or "dube spray" or "duncaine" or "dynexan" or "ela-max" or "esracain" or "farmacaina" or "gesicain" or "glydo" or "gravocain" or "isicaine" or "jetokain" or "l-caine" or "lecasin" or "leostesin" or "lida mantle" or "lidocaton" or "lidocor" or "lidocorit" or "lidoderm" or "lidoject" or "lidonest" or "lidopain" or "lidopen" or "lidothesin" or "lignocaine" or "lignostab" or "lincaine" or "liquocaine" or "maricaine" or "n,n diethyl n' (2,6 dimethylphenyl)glycinamide" or "neo novutox" or "neolidocaton" or "omega diethylamino 2,6 dimethylacetanilide" or "octocaine" or "otipax" or "remicaine" or "restylane lyft" or "roxicaina" or "rucaina" or "ruciana" or "solarcaine" or "solcaine" or "truxacaine" or "uad caine" or "vasocaine" or "versatis" or "xidocaine" or "xiline" or "xilocaina" or "xilonest" or "xilotane" or "xilyne" or "xylcaine" or "xylestesin" or "xylesthesin" or "xylocain*" or "xylocard" or "xylocitin" or "xyloctin" or "xyloneural" or "xylonor" or "xyloproct" or "xyloton" or "xylotox" or "xylyne" or "zingo" or "ztlido").ab,kf,ti.) AND (exp Cardiac Surgical Procedures/ or exp Sternotomy/ OR ((cardiosurg* or ((aortocoronary or cardi* or intracardi* or pericardi* or coronary or heart or myocardi* or transmyocardi* or valv* or internal-mammary) adj3 (anastomosis or annul* or bypass* or resection or revasculari* or surg* or operation* or recanali* or shunt* or reperfusion* or stenting or aneurysmectom* or massage or graft* or transplant* or allograft* or heterograft* or allotransplant* or heterotransplant* or homograft* or homotransplant* or xenotransplant* or implant* or atherectom* or balloon-dilation)) or sternotom* or sternum-osteotom* or ((atrial or arterial or double) adj1 switch) or jatene or rastelli or senning or atrial-switch or mustard or cardiomyoplast* or annuloplast* or (valve adj2 annul*) or angioplast* or vineberg or fontan or hemi-fontan or norwood or glenn or ((bidirectional or bi-directional or cavopulmonary) adj2 (shunt* or anastomos*)) or pericardiostom* or pericardial-window or pericardiectom* or pericardotom* or pericardiotom* or pericardiocentesis or pericardicentesis or (pericardi* adj2 (aspirat* or punctur*)) or ((valv* or bicuspid or tricuspid) adj2 (bioprosthe* or prosthe* or replac* or artificial or repair or reconstruct*)) or valvuloplast* or valvulotom* or valvotom* or commissurotom* or ross).ab,kf,ti.) AND (exp Postoperative Care/ or exp Postoperative Complications/ or exp Postoperative Period/ OR ((postoperative* or post-operative* or post-op* or postsurg* or post-surg* or post-procedur* or postprocedur* or postanesth* or postanaesth* or post-anesth* or post-anaesth* or PONV).ab,kf,ti.) OR exp "Length of Stay"/ OR ("length of stay" or "hospital stay*" or "stay length*" or LOS).ab,kf,ti.)) NOT (exp CHILD/ not exp ADULT/, exp Animals/ not Humans/) Limit to 1980+

**Scopus:**(TITLE-ABS-KEY(lidocaine or "2-(diethylamino)-n-(2,6-dimethylphenyl)acetamide" or "2-2etn-2mephacn" or "2 diethylamino 2',6' acetoxylidide" or aeroderm or akten or "alpha diethylamino 2,6 dimethylacetanilide" or alphacaine or anestacaine or anestacon* or aritmal or astracaine or betacaine or cidancaina or "corus 1030" or "corus1030" or cuivasil or dalcaine or dentipatch or dequaspray or "diethylamino 2,6 dimethylacetanilide hydrochloride" or diethylaminodimethylacetanilid* or dolicaine or "dube spray" or duncaine or dynexan or ela-max or esracain or farmacaina or gesicain or glydo or gravocain or isicaine or jetokain or l-caine or lecasin or leostesin or "lida mantle" or lidocaton or lidocor or lidocorit or lidoderm or lidoject or lidonest or lidopain or lidopen or lidothesin or lignocaine or lignostab or lincaine or liquocaine or maricaine or "n,n diethyl n' (2,6 dimethylphenyl)glycinamide" or "neo novutox" or "neolidocaton" or "omega diethylamino 2,6 dimethylacetanilide" or octocaine or otipax or remicaine or "restylane lyft" or roxicaina or rucaina or ruciana or solarcaine or solcaine or truxacaine or "uad caine" or vasocaine or versatis or xidocaine or xiline or xilocaina or xilonest or xilotane or xilyne or xylcaine or xylestesin or xylesthesin or xylocain* or xylocard or xylocitin or xyloctin or xyloneural or xylonor or xyloproct or xyloton or xylotox or xylyne or zingo or ztlido)) AND (TITLE-ABS-KEY ( cardiosurg*  OR  ( ( aortocoronary  OR  cardi*  OR  intracardi*  OR  pericardi*  OR  coronary  OR  heart  OR  myocardi*  OR  transmyocardi*  OR  valv*  OR  internal-mammary )  W/3  ( anastomosis  OR  annul*  OR  bypass*  OR  resection  OR  revasculari*  OR  surg*  OR  operation*  OR  recanali*  OR  shunt*  OR  reperfusion*  OR  stenting  OR  aneurysmectom*  OR  massage  OR  graft*  OR  transplant*  OR  allograft*  OR  heterograft*  OR  allotransplant*  OR  heterotransplant*  OR  homograft*  OR  homotransplant*  OR  xenotransplant*  OR  implant*  OR  atherectom*  OR  balloon-dilation ) )  OR  sternotom*  OR  sternum-osteotom*  OR  ( ( atrial  OR  arterial  OR  double )  W/1  switch )  OR  jatene  OR  rastelli  OR  senning  OR  atrial-switch  OR  mustard  OR  cardiomyoplast*  OR  annuloplast*  OR  ( valve  W/2  annul* )  OR  angioplast*  OR  vineberg  OR  fontan  OR  hemi-fontan  OR  norwood  OR  glenn  OR  ( ( bidirectional  OR  bi-directional  OR  cavopulmonary )  W/2  ( shunt*  OR  anastomos* ) )  OR  pericardiostom*  OR  pericardial-window  OR  pericardiectom*  OR  pericardotom*  OR  pericardiotom*  OR  pericardiocentesis  OR  pericardicentesis  OR  ( pericardi*  W/2  ( aspirat*  OR  punctur* ) )  OR  ( ( valv*  OR  bicuspid  OR  tricuspid )  W/2  ( bioprosthe*  OR  prosthe*  OR  replac*  OR  artificial  OR  repair  OR  reconstruct* ) )  OR  valvuloplast*  OR  valvulotom*  OR  valvotom*  OR  commissurotom*  OR  ross ) ) AND ( TITLE-ABS-KEY ( postoperative*  OR  post-operative*  OR  post-op*  OR  postsurg*  OR  post-surg*  OR  post-procedur*  OR  postprocedur*  OR  postanesth*  OR  postanaesth*  OR  post-anesth*  OR  post-anaesth*  OR  ponv )  OR  TITLE-ABS-KEY ( "length of stay"  OR  "hospital stay*"  OR  "stay length*"  OR  los ) )  Limit to 1980+

**Web of Science:**

TS=(lidocaine or "2-(diethylamino)-n-(2,6-dimethylphenyl)acetamide" or "2-2etn-2mephacn" or "2 diethylamino 2',6' acetoxylidide" or aeroderm or akten or "alpha diethylamino 2,6 dimethylacetanilide" or alphacaine or anestacaine or anestacon* or aritmal or astracaine or betacaine or cidancaina or "corus 1030" or "corus1030" or cuivasil or dalcaine or dentipatch or dequaspray or "diethylamino 2,6 dimethylacetanilide hydrochloride" or diethylaminodimethylacetanilid* or dolicaine or "dube spray" or duncaine or dynexan or ela-max or esracain or farmacaina or gesicain or glydo or gravocain or isicaine or jetokain or l-caine or lecasin or leostesin or "lida mantle" or lidocaton or lidocor or lidocorit or lidoderm or lidoject or lidonest or lidopain or lidopen or lidothesin or lignocaine or lignostab or lincaine or liquocaine or maricaine or "n,n diethyl n' (2,6 dimethylphenyl)glycinamide" or "neo novutox" or "neolidocaton" or "omega diethylamino 2,6 dimethylacetanilide" or octocaine or otipax or remicaine or "restylane lyft" or roxicaina or rucaina or ruciana or solarcaine or solcaine or truxacaine or "uad caine" or vasocaine or versatis or xidocaine or xiline or xilocaina or xilonest or xilotane or xilyne or xylcaine or xylestesin or xylesthesin or xylocain* or xylocard or xylocitin or xyloctin or xyloneural or xylonor or xyloproct or xyloton or xylotox or xylyne or zingo or ztlido) AND

TS=(cardiosurg* OR (( aortocoronary OR cardi* OR intracardi* OR pericardi* OR coronary OR heart OR myocardi* OR transmyocardi* OR valv* OR internal-mammary) NEAR/3 ( anastomosis OR annul* OR bypass* OR resection OR revasculari* OR surg* OR operation* OR recanali* OR shunt* OR reperfusion* OR stenting OR aneurysmectom* OR massage OR graft* OR transplant* OR allograft* OR heterograft* OR allotransplant* OR heterotransplant* OR homograft* OR homotransplant* OR xenotransplant* OR implant* OR atherectom* OR balloon-dilation )) OR sternotom* OR sternum-osteotom* OR ((atrial OR arterial OR double ) NEAR/1 switch ) OR jatene OR rastelli OR senning OR atrial-switch OR mustard OR cardiomyoplast* OR annuloplast* OR (valve NEAR/2 annul*) OR angioplast* OR vineberg OR fontan OR hemi-fontan OR norwood OR glenn OR ((bidirectional OR bi-directional OR cavopulmonary) NEAR/2 (shunt* OR anastomos*)) OR pericardiostom* OR pericardial-window OR pericardiectom* OR pericardotom* OR pericardiotom* OR pericardiocentesis OR pericardicentesis OR (pericardi* NEAR/2 (aspirat* OR punctur*)) OR (( valv* OR bicuspid OR tricuspid ) NEAR/2 (bioprosthe* OR prosthe* OR replac* OR artificial OR repair OR reconstruct*)) OR valvuloplast* OR valvulotom* OR valvotom* OR commissurotom* OR ross) AND TS=(postoperative* or post-operative* or post-op* or postsurg* or post-surg* or post-procedur* or postprocedur* or postanesth* or postanaesth* or post-anesth* or post-anaesth* or PONV OR "length of stay" or "hospital stay*" or "stay length*" or LOS) Limt to 1980+
